# Clinical characteristics of psychogenic pseudosyncope in a population-based cohort

**DOI:** 10.1093/europace/euaf125

**Published:** 2025-06-21

**Authors:** Karl Firth, Isabella Kharraziha, Madeleine Johansson, Richard Sutton, Daiva Daukantaité, Viktor Hamrefors, Artur Fedorowski

**Affiliations:** Department of Clinical Sciences, Lund University, Jan Waldenströms gata 35, Malmö 214 28, Sweden; Department of Cardiology, Skåne University Hospital, Jan Waldenströms gata 35, Malmö 214 28, Sweden; Department of Clinical Sciences, Lund University, Jan Waldenströms gata 35, Malmö 214 28, Sweden; Department of Cardiology, Skåne University Hospital, Jan Waldenströms gata 35, Malmö 214 28, Sweden; Department of Clinical Sciences, Lund University, Jan Waldenströms gata 35, Malmö 214 28, Sweden; Department of Cardiology, Skåne University Hospital, Jan Waldenströms gata 35, Malmö 214 28, Sweden; National Heart and Lung Institute, Imperial College, London, UK; Department of Cardiology, Hammersmith Hospital, London, UK; Department of Psychology, Lund University, Lund, Sweden; Department of Clinical Sciences, Lund University, Jan Waldenströms gata 35, Malmö 214 28, Sweden; Department of Cardiology, Skåne University Hospital, Jan Waldenströms gata 35, Malmö 214 28, Sweden; Department of Clinical Sciences, Lund University, Jan Waldenströms gata 35, Malmö 214 28, Sweden; Department of Cardiology, Skåne University Hospital, Jan Waldenströms gata 35, Malmö 214 28, Sweden; Department of Cardiology, Karolinska University Hospital, Stockholm, Sweden; Department of Medicine, Karolinska Institute, Stockholm, Sweden

**Keywords:** Psychogenic pseudosyncope, Vasovagal syncope, Tilt-table test, Accidental falls, Psychotropic drugs, Medical history taking

What’s new?

- Patients diagnosed with psychogenic pseudosyncope reported significantly higher rates of supine syncope, syncopal episodes, and traumatic falls than patients diagnosed with vasovagal syncope in a survey questionnaire completed before head-up tilt test.

Apparent transient loss of consciousness (TLOC) with the absence of abnormal changes in blood pressure or heart rate is referred to as psychogenic pseudosyncope (PPS).^1,2^ Whilst vasovagal syncope (VVS) can be triggered by salient emotional events, which may also be considered psychological in origin, it differs from PPS in that it leads to cardiovascular changes that impact cerebral perfusion.^1^ A detailed medical history and head-up tilt test (HUTT) with apparent loss of consciousness in the absence of abnormal changes in blood pressure or heart rate are required for positive diagnosis of PPS.^3^

This study compared PPS and VVS patients to facilitate differential diagnosis. We performed retrospective analysis of patients referred for unexplained syncope between 2008 and 2021 to the tertiary syncope unit, Skåne University Hospital, Malmö, Sweden, using the previously described SYSTEMA database of 2690 patients.^4^ The database has 168 variables describing demographics, fainting history, further medical history (including drug therapy), HUTT haemodynamic details, blood tests result, and final adjudicated diagnosis.^5^

A 21-item questionnaire assisted medical history taking. Diagnosis of PPS and VVS followed current European Society of Cardiology syncope guidelines, i.e. reproduction of PPS during HUTT with typical features (closed eyes, loss of responsiveness, prolonged duration after being tilted down, and absence of abnormal changes in blood pressure and heart rate).^1^ Patients with PPS and a positive diagnosis of either postural orthostatic tachycardia syndrome (POTS), VVS, or orthostatic hypertension identified during HUTT (regardless of temporal sequence relative to PPS) were classified as multiple aetiology TLOC.

Seventy-eight patients (2.9%) were diagnosed as PPS (29 multiple TLOC aetiology: POTS 14; VVS 18; both 3). Clinical characteristics of PPS and VVS patients are summarized in *Table 1*. The percentage of females was 85.9% in the PPS group vs. 62.6% for VVS % [risk ratio (RR) of 1.37, 95% confidence interval (CI) 1.24–1.51, *P* < 0.001]. Patients diagnosed with PPS were significantly younger having a median age of 30 years [interquartile range (IQR) *=* 18.3], whilst the VVS cohort was 47 years (IQR = 35.2, *P* < 0.001). Median number of syncopal episodes reported by PPS and VVS patients was 30 (IQR = 88) and 5 (IQR = 8, *P* < 0.001), respectively.

**Table 1:** Clinical characteristics of patients with PPS compared with reflex VVS

Clinical feature	PPS	VVS	Inc. risk ratio (95%CI)	*P*-value
Diagnosis, *n* (%)	78 (2.9)	1459 (54.2)	—	—
Age, mean (SD)	34.8 (13.5)	46.9 (19.9)	—	<0.001
Time since first syncope, mean (SD)	7.7 (8)	11.4 (14.9)		<0.001
Female, *n* (%)	67 (85.9)	913 (62.6)	1.37 (1.24–1.51)	<0.001
Supine HR, mean (SD)	72.1 (11.9)	68.9 (11.4)	—	0.022
Supine SBP, mean (SD)	123.9 (18.7)	131.2 (20.2)	—	0.001
Prodromes, *n* (%)	49 (62.8)	829 (56.8)	1.10 (0.92–1.31)	0.372
Palpitations, *n* (%)	34 (43.6)	336 (23)	1.88 (1.44–2.47)	<0.001
Supine syncope, *n* (%)	49 (62.8)	263 (18.1)	3.48 (2.84–4.26)	<0.001
Traumatic falls, *n* (%)	57 (73.1)	732 (50.2)	1.45 (1.26–1.68)	<0.001
Dizziness on standing, *n* (%)	66 (84.6)	984 (67.4)	1.25 (1.13–1.39)	0.002
Frequency of episodes, median (IQR)	30 (88)	5 (8)	—	<0.001
BMI, mean (SD)	25 (5.46)	25.1 (4.28)	—	0.142
Smoker, *n* (%)	16 (20.5)	169 (11.6)	1.78 (1.13–2.82)	0.017
Antidepressants, *n* (%)	16 (20.5)	153 (10.5)	1.95 (1.23–3.09)	0.006
Sedatives, (inc. benzo) *n* (%)	8 (10.3)	58 (4)	2.57 (1.27–5.19)	0.018
Sleep agents, *n* (%)	10 (12.8)	57 (3.9)	3.27 (1.74–6.15)	<0.001
Examinations prior to evaluation at syncope unit				
Brain CT/MRI *n* (%)	61 (78.2)	782 (53.9)	1.45 (1.28–1.65)	<0.001
EEG, *n* (%)	44 (54.4)	426 (29.2)	1.92 (1.56–2.37)	<0.001
Neuro. consultation, *n* (%)	34 (43.6)	293 (20.1)	2.16 (1.65–2.84)	<0.001
Holter ECG, *n* (%)	57 (73.1)	841 (57.6)	1.26 (1.10–1.45)	0.008

Continuous variables are presented as mean ± standard deviation (SD) for normally distributed data or median ± interquartile range (IQR) for non-normally distributed data. Dichotomous data are expressed as percentages within each group. *P*-values indicate differences between groups, using independent samples *t*-test for normally distributed continuous variables, the Wilcoxon rank-sum test for non-normally distributed continuous variables, and the *χ*^2^ test for dichotomous data. The relative risk ratio was calculated using the epiR package for *R*. Patients diagnosed with both PPS and VVS were classified as PPS (*n* = 18).

BMI, body mass index; CT, computed tomography; ECG, electrocardiography; EEG, electroencephalography; HR, heart rate; MRI, magnetic resonance imaging; PPS, psychogenic pseudosyncope; SBP, systolic blood pressure; VVS, vasovagal syncope.

Significantly, more patients with PPS reported supine syncope than those with VVS (68.82% vs. 18.05%, *P* < 0.001), with an increased RR of 3.48, 95% CI 2.84–4.26. Patients with PPS also reported significantly higher occurrences of traumatic falls than VVS patients (73.08% vs. 50.38%, *P* < 0.001, RR of 1.45, 95% CI 1.26–1.68).

To differentiate better between VVS and PPS diagnoses, a logistic regression model was employed to assess the relationship between potential predictors and the likelihood of a PPS diagnosis. Age, gender, frequency of episodes, history of fall trauma, and supine syncope were included as independent variables. The analysis included 1504 observations after excluding 33 cases with missing data. The model achieved a McFadden’s *R*² of 0.2216, indicating that 22.16% of the variability in the diagnosis was explained by the predictors. This represents a good fit for logistic regression, although additional established clinical features of PPS not captured in this model may further enhance diagnostic accuracy. A receiver operating characteristic curve demonstrated excellent discriminatory power, with an area under the curve of 0.85 (95% CI 0.8059–0.8933) indicating a strong ability to distinguish between PPS and VVS. Supine syncope was the strongest predictor, with an odds ratio of 4.86 (95% CI 2.84–8.43), highlighting its importance in identifying PPS cases. No collinearity was detected, with variable inflation factors ranging from 1.02 to 1.06.

The analysis of a large database of patients unexplained syncope patients shows ∼3% of patients were diagnosed with PPS. Patients were mainly women, significantly younger than the total cohort, and reported significantly more fainting episodes than those with VVS. Patients with PPS were more likely to report both traumatic falls, palpitations, and supine syncope. Whilst there were similarities in the profile of the VVS and PPS groups in terms of age, gender, report of the first syncopal event, and reports of palpitations, there is a clear delineation between the two groups in terms of the frequency of events, supine syncope, and traumatic falls. We are unable to explain the higher rate of traumatic falls observed in PPS patients compared with VVS patients.

The upright position promotes syncope because of gravitational effects. Although syncope in the supine position has been observed in late pregnancy,^6^ it is rare in non-pregnant subjects,^7^ usually associated with cardiac arrhythmia and should prompt swift cardiac evaluation.^1^

Previous studies have reported overlap between VVS and PPS.^8,9^ The study of Blad *et al*. highlights differences of 23 patients exhibiting signs of both and compares them to a matched control group of VVS only. They found an increased likelihood ratio of atypical triggers in the PPS/VVS group compared with VVS only [( + LR) 5.00, 95% CI 2.04–12.24]. Atypical triggers are defined as exercise or TLOC in the supine position.^8^ Whilst Walsh *et al*.^9^ do not mention supine syncope directly, they note the lack of typical VVS triggers in the PPS group. Although supine apparent syncope related to emotional triggers has been noted,^10^ its incidence in PPS patients is not previously reported.

The main limitation of these findings is that the data are single centre. However, the centre serves a large area of Sweden and the patient population can be considered relatively diverse. Furthermore, the findings relating to supine syncope and traumatic falls come from self-report questionnaires. No further information is available on hospital admissions, injuries, and fall frequency. However, the strengths of the study come from both the large numbers of completed questionnaires and the association between supine syncope, traumatic falls, and PPS.

We found that PPS patients are predominantly women and significantly younger than most patients referred for HUTT. They report more fainting episodes and traumatic falls and undergo more examinations than VVS patients. A new finding that PPS patients report significantly higher levels of supine syncope is an important aspect to add to medical history taking, either directly or through a questionnaire instrument.

## Data Availability

The data underlying this article cannot be shared publicly due to legislation regarding data protection. However, the data may be shared upon reasonable request to the principal investigator.
